# Possible role of autophagy induced by COVID-19 in cancer progression, chemo-resistance, and tumor recurrence

**DOI:** 10.1186/s13027-022-00450-2

**Published:** 2022-07-18

**Authors:** Hamidreza Zalpoor, Abdullatif Akbari, Negar Nayerain Jazi, Mahsa Liaghat, Maryam Bakhtiyari

**Affiliations:** 1 Shiraz Neuroscience Research Center, Shiraz University of Medical Sciences, Shiraz, Iran; 2 Network of Immunity in Infection, Malignancy and Autoimmunity (NIIMA), Universal Scientific Education & Research Network (USERN), Tehran, Iran; 3 Department of Bacteriology and Virology, School of Medicine, Shiraz University of Medical Sciences, Shiraz, Iran; 4 Department of Medical Laboratory Sciences, Faculty of Medical Sciences, Kazerun Branch, Islamic Azad University, Kazerun, Iran; 5 Department of Medical Laboratory Sciences, Faculty of Allied Medicine, Qazvin University of Medical Sciences, Qazvin, Iran

**Keywords:** COVID-19, SARS-CoV-2, Autophagy, Cancer progression, Tumor recurrence, Chemo-resistance

## Abstract

COVID-19 infection is a serious threat to patients with primary diseases, especially multiple cancers. Studies suggest that cancer patients are one of the most susceptible populations to experience severe COVID-19 and death. In addition, a number of studies suggest various mechanisms for SARS-CoV-2 in cancer progression. In this study, we discussed the role of SARS-CoV-2 in the induction of autophagy and we hypothesized that autophagy induced by COVID-19 not only can contribute to viral replication but also potentially can lead to cancer progression, chemo-resistance, and tumor recurrence in multiple cancer patients. Therefore, targeting autophagy-related signaling pathways and cellular and molecular processes could be a potentially promising therapeutic approach for cancer patients with COVID-19. Hence, this study can shed light on a new window on the management of such patients. However, more investigations in the future are required to understand other pathological effects of COVID-19 infection on cancer patients to provide new therapeutic strategies to combat these complications in these patients.

## Dear Editor,

Nowadays, Coronavirus disease-2019 (COVID-19) pandemic which caused by severe acute respiratory syndrome coronavirus 2 (SARS-CoV-2) has been a serious issue worldwide. It has been suggested that patients with cancer are one of the major cases who are susceptible to experience severe COVID-19 courses and death, potential long-term impact of COVID-19 on cancer patients' prognosis and progression, as well as their response to different anti-cancer therapies [1–5]. SARS-CoV-2 can exert its role by induction of autophagy to lead to these COVID-19 complications [6, 7].

Here, we hypothesized that COVID-19 by induction autophagy in tumor cells not only can lead to cancer progression but also can lead to chemo-resistance and tumor recurrence in these patients. Therefore, pharmacological targeting autophagy for cancer patients with COVID-19 must be considered in future investigations.

**Fig. 1:**
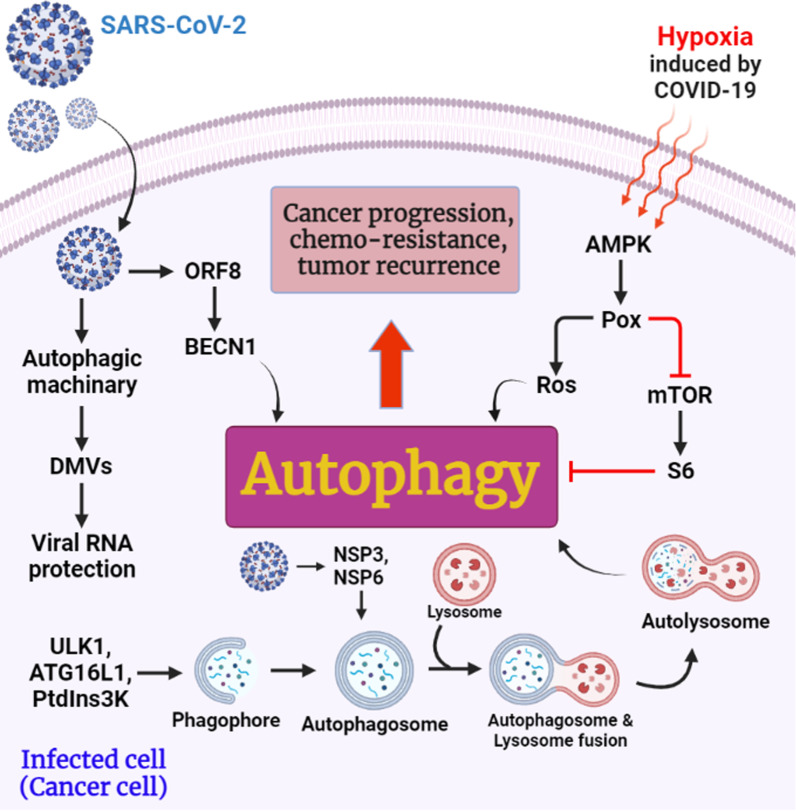
Potential role of SARS-CoV-2 in the induction of autophagy in infected cells (cancer cells), which can lead to cancer progression, chemo-resistance, and tumor recurrence

Autophagy is a preserved cellular process that involves enclosing cytoplasmic residues such as aged proteins, and organelles in autophagosomes, and delivering them to lysosomes for degradation [6]. Multiple steps are involved in the autophagy pathway. Initially, the phagophore, the compartment that sequesters autophagy, originates and expands. An autophagosome is formed when the phagophore closes and traps cargo in its double membrane [6]. ULK1 complex, PtdIns3K complex, and autophagy-related (ATG)16L1 complex are involved in the formation of autophagosomes [7] (Fig. 1). As the autophagosome merges with the endosome, the acidic amphisome is formed. As a final step, the amphisome merges with the lysosome to facilitate the degradation of vesicular contents in the autolysosome [6].

Interestingly, the process of autophagy is induced by many Coronaviruses, even though a few of these viruses arrest its progression before autophagic degradation. On the other hand, some coronaviruses seize autophagy pathway components in a non-canonical way [6]. However, the interaction between Coronaviruses and autophagy is extremely complicated and not fully understood. Coronaviruses utilize autophagy for viral replication. They may induce distinctive rearrangements in endoplasmic reticulum (ER) membranes that lead to the formation of intricate membranes and connected double membrane vesicles (DMVs). These cytosolic membranes function as a scaffold for viral RNA and protect viral elements from the host cells' defensive mechanisms [7] (Fig. 1). In a recent study, it was demonstrated that the protein encoded by SARS-CoV-2 ORF8 promotes lysosomal degradation of the major histocompatibility complex I (MHC-I) molecules through an autophagy pathway dependent on BECN1 [7]. Another study confirmed that β-coronaviruses (β-CoV) induced autophagosome formation via NSP6 mediated ATG5 [8]. The levels of SQSTM1 increases approximately 1.5-fold when SARS-CoV-2 ORF7a, ORF3a, and E proteins are present [7]. ATG8 proteins bind ubiquitylated targets and direct them to autophagosomes through autophagy receptors, such as SQSTM1/p62.

Mouse hepatitis virus (MHV, a β-CoV), NSP3 of CoV-NL63, and NSP6 of infectious bronchitis virus (IBV, a γ-coronavirus) develop autophagosomes formation, which is often associated with virus replication complexes. Although, Coronavirus proteins can stimulate DMVs directly from the ER, including NSP3, 4 and 6 of SARS-CoV-1, and NSP3 and 4 of Middle East Respiratory Syndrome Coronavirus (MERS-CoV) [6]. IBV infection leads to autophagy through ERN1/IRE1, which is one of the unfolded protein response pathways (UPR) involved in autophagy through ER stress [7].

Coronaviruses can also interfere with the fusion of autophagosomes and lysosomes. Fusion can be blocked by directly or indirectly inhibiting BECN1, a host protein that promotes fusion. The PLP-domain of CoV-NL63 NSP3 binds STING1 and BECN1, inhibiting BECN1's effects on autophagosome and lysosome fusions and inhibiting interferon production [6]. MERS-CoV infection prevents autophagy through NSP6 and accessory proteins 4b and 5 at the stage of autolysosome construction [7].

Interestingly, in addition to the fact that the SARS-CoV-2 virus can directly induce autophagy, the conditions it causes can also contribute to autophagy. These conditions include hypoxia, oxidative stress, and the production of inflammatory cytokines.

In a previous study, we suggested that hypoxia induced by SARS-CoV-2 infection can lead to activation of both (HIF-1α) and autophagy, which could be a hallmark for acute myeloid leukemia (AML) patients and leukemia progression, especially for AML patients who have Flt3-ITD (FMS-like tyrosine kinase-3 receptor internal tandem duplications) mutations [5].

In addition, according to Mehri et al. studies, it has been shown that the amount of reactive oxygen species (ROS) in COVID-19 patients is directly related to the disease burden. Respiratory macrophages have been shown to produce significant amounts of ROS in response to SARS-CoV-2 infection. NADPH oxidase 2 (NOX2), which plays a significant role in the production of ROS, is also found in high levels in the serum of these patients. Hypoxia caused by infection can cause the production of H2O2, which is involved in the overproduction of inflammatory cytokines. Also, ROS activates NLRP3/NALP3 and triggers a cytokine storm by activating the inflammasome. In addition, ROS can increase inflammasomes by activating NF-κB [9].

Accordingly, Martinez-Outschoorn, Whitaker-Menezes’s studies performed on co-cultured fibroblasts with cancer cells, and the treatment of these cells with inflammatory factors such as IL-6, IL-8, IFN-γ, RANTES, and TNF-α cause an increase in LC3B -II as an autophagic response marker. Also, according to the in vitro study of this group, the oxidative stress imposed on fibroblast cells in the microenvironment of breast cancer tumors, leads to the production of inflammatory cytokines through the activation of NF-κB and ultimately activates inflammation and autophagy. Autophagy causes the loss of Caveolin-1 (CAV-1) in these cells and this condition intensifies oxidative stress and autophagy and leads to tumor recurrence [10].

Importantly, according to a study by Liu et al. Hypoxia causes upregulation of AMPK in the hypoxic tumor microenvironment (TME) occurs in many cancers, including colon, renal, breast, prostate, melanoma, lung, and ovarian cancer. This study showed that AMP-activated protein kinase (AMPK) can cause proline oxidase (POX) overexpression as an enzyme that converts proline into an energy source under stress conditions. POX also induces autophagy through 2 pathways. (a) Increases ROS levels in TME, and (b): induces autophagy by inhibiting mTOR (as an autophagy suppressor) and its downstream factor S6 [11] (Fig. 1).

Autophagy can act as a tumor enhancer in some cases by maintaining cellular homeostasis in nutrient-depleted and hypoxic conditions [12]. There are cancer cells that remain in a "dormant" state between tumor formation and recurrence (also known as relapse), demonstrating balanced proliferation or no proliferation at all during this time. Dormant cells can sometimes reactivate and develop new metastatic lesions. Such tumors tend to be drug-resistant and aggressive. Therefore, the prognosis of most patients with recurrent disease is poor [8]. Thus, it seems that SARS-CoV-2 potentially can promote dormant cells by activating autophagy pathways.

Studies have demonstrated that autophagy enhances tumor cell survival and leads to therapy resistance in some cancer types, such as breast cancer, prostate cancer, and gastrointestinal stromal tumors. Additionally, autophagy has been implicated in protecting cancer stem cells in pancreatic cancer, glioblastoma, colorectal cancer, chronic myeloid leukemia, and bladder cancer [13].

Kim et al. provided the first evidence for the function of breast cancer cell-astrocyte interactions in driving brain metastatic transformation through the up-regulation of several pro-survival genes, including those stimulating autophagy. Increased resistance to various chemotherapeutics is linked to these cell–cell interactions and cancer cell survival [14]. In addition, Lee et al. discovered that a new autophagy regulator causes tamoxifen resistance in breast cancer cells [14].

According to Ojha et al. [12], a high autophagic rate in side population cells is linked to resistance to chemotherapy. As a result, autophagy is linked to cell survival in bladder cancer and could be a promising target for creating more effective treatments to improve patient survival. In addition, conventional methods such as radiotherapy and cytotoxic chemotherapy kill the dividing cells in cancer stem cells (CSCs), but the tumor-initiating CSCs are alive due to an autophagy-mediated cell survival mechanism. Consequently, inhibiting autophagy in CSCs could reduce tumor resistance and recurrence. Other studies show that autophagy may be a contributing element in the reported robustness of dormant cells when confronted with anticancer treatments, as autophagic activity is enhanced in dormant cells [8].

In recent studies, autophagy-related drugs have been suggested as potential anti-SARS-CoV-2 agents, from numerous in vitro to in vivo studies [15].

In conclusion, this evidence suggests that COVID-19 can stimulate autophagy by using multiple factors and we hypothesized that autophagy induced by COVID-19 potentially can contribute to cancer progression, chemo-resistance, and tumor recurrence in cancer patients with COVID-19. Hence, targeting autophagy not only can be an anti-viral therapeutic strategy but also can be a promising therapeutic approach for cancer patients with COVID-19 to reduce the risk of mortality, cancer progression, chemo-resistance, and tumor recurrence. We encourage researchers to conduct new studies to confirm this hypothesis.

## Data Availability

Data sharing is not applicable to this article as no new data were created or analyzed in this study.

## Abbreviations

COVID-19: Coronavirus disease-2019
ATG: Autophagy-related gene
SARS-CoV-2: Severe acute respiratory syndrome coronavirus 2
ER: Endoplasmic reticulum
DMVs: Double membrane vesicles
β-CoV: β-Coronaviruses
TME: Tumor microenvironment
NSP: Non-structural proteins
